# ESO annual stroke evidence update 2025

**DOI:** 10.1093/esj/aakag009

**Published:** 2026-02-17

**Authors:** Diana Aguiar de Sousa, Aristeidis H Katsanos, Linxin Li, Nicolas Martinez-Majander, John McCabe, Lina Palaiodimou, Anna Ramos Pachon, Michele Romoli, Peter D Schellinger, Annaelle Zietz, Sven Poli, Melinda Roaldsen, Ashkan Shoamanesh, Marek Sýkora, Georgios Tsivgoulis

**Affiliations:** Stroke Center, Department of Neurosciences, ULS São José, Lisbon, Portugal; Faculdade de Medicina, Universidade de Lisboa, Lisbon, Portugal; Gulbenkian Institute for Molecular Medicine, Lisbon, Portugal; Division of Neurology, McMaster University/Population Health Research Institute, Hamilton, Ontario, Canada; Wolfson Centre for Prevention of Stroke and Dementia, Nuffield Department of Clinical Neurosciences, University of Oxford, Oxford, United Kingdom; Department of Neurology, Helsinki University Hospital and University of Helsinki, Helsinki, Finland; Stroke Service, Department of Geriatric Medicine, Mater Misericordiae University Hospital, Dublin, Ireland; Second Department of Neurology, Medical School, National and Kapodistrian University of Athens, “Attikon” University Hospital, Athens, Greece; Stroke Unit, Department of Neurology, Hospital de la Santa Creu i Sant Pau, Barcelona, Spain; Department of Neurosciences, Neurology and Stroke Unit, Bufalini Hospital, AUSL Romagna, Cesena, Italy; Department of Neurology and Neurogeriatrics, John Wesling Medical Center Minden, UK RUB, Minden, Germany; Department of Neurology and Stroke Centre, University Hospital Basel and University of Basel, Basel, Switzerland; Department of Neurology and Stroke and Hertie Institute for Clinical Brain Research, University of Tübingen, Tübingen, Germany; Clinical Research Department, University Hospital of North Norway, Tromsø, Norway; Division of Neurology, McMaster University/Population Health Research Institute, Hamilton, Ontario, Canada; Department of Neurology, St. John’s Hospital, Sigmund Freud University, Vienna, Austria; Second Department of Neurology, Medical School, National and Kapodistrian University of Athens, “Attikon” University Hospital, Athens, Greece

**Keywords:** stroke, update, 2025, recommendations, reperfusion therapies, secondary prevention, rehabilitation

## Abstract

**Background:**

Stroke medicine is evolving rapidly, with emerging evidence continuously informing clinical practice. The European Stroke Organisation (ESO) Annual Stroke Evidence Update is a new ESO Guideline Board initiative that bridges the interval between formal guideline revisions by providing a curated synthesis of recent advances that may inform stroke care.

**Methods:**

This edition synthesises new evidence published or presented during 2025 across various key domains, including acute treatment, prevention, rehabilitation, imaging, systems of care and outcomes. We searched major stroke and internal medicine journals and reviewed abstract proceedings from the main international stroke conferences. Studies were shortlisted by domain leads and selected by consensus among ESO Guideline Board members based on methodological quality, clinical relevance and potential to impact stroke management.

**Results:**

Three randomised trials found no benefit for endovascular thrombectomy over best medical therapy in stroke due to MeVO. One trial showed that intravenous tenecteplase before thrombectomy was associated with improved functional independence at 90 days compared with thrombectomy alone in patients with stroke due to LVO. Individual patient data meta-analysis of 4 trials confirmed that intensive blood pressure lowering in the acute phase of ICH was safe and improved functional recovery, with greatest benefit when started within 3 h. A trial showed that triple-pill combination therapy achieved blood pressure control and reduced recurrent stroke after ICH. For ischaemic stroke associated with atrial fibrillation an individual participant data meta-analysis of 4 trials confirmed that early direct oral anticoagulant initiation within 4 days reduced recurrent ischaemic stroke without increasing symptomatic ICH. In non-cardioembolic stroke and high risk TIA, asundexian, a novel factor XIa inhibitor, reduced ischaemic stroke risk without increasing major bleeding in a large phase 3 trial. Mobile stroke units were shown to be cost-effective in high-volume systems. Promising therapeutic strategies for post-stroke cognitive impairment include brain stimulation, computerised cognitive training and cardiorespiratory training.

**Conclusions:**

In 2025, trial evidence supported no routine role for EVT in MeVO, reinforced tenecteplase as bridging thrombolysis in eligible LVO patients undergoing EVT and strengthened the signal for early intensive blood pressure lowering in acute ICH. Priority research questions include identifying subgroups that may benefit from EVT in MeVO, defining the role of adjunct intra-arterial thrombolysis after successful EVT and clarifying which imaging-based selection pathways are most feasible and effective across different stroke systems.

## Graphical Abstract

**Graphical Abstract:**
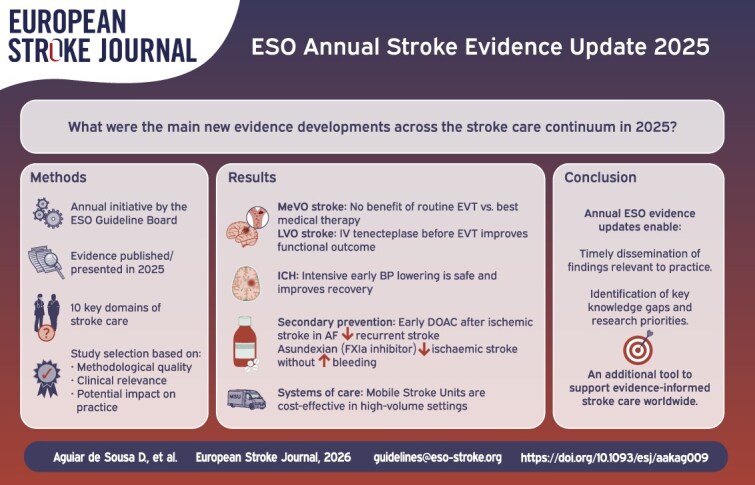

## Introduction

Stroke medicine continues to advance rapidly, with emerging evidence informing clinical practice and future evidence-based guidelines. The ESO Stroke Evidence Update is a new initiative from the European Stroke Organisation (ESO) Guideline Board, designed to provide a timely overview of recent advances and to bridge the gap between formal guideline revisions. Accordingly, where practical implications are discussed, these reflect the authors’ expert interpretation of the current evidence and do not constitute guideline recommendations.

This first edition synthesises key evidence published or presented during 2025, focusing on findings with potential implications for day-to-day stroke care and future ESO guideline development. The review encompasses key domains across acute treatment, prevention, rehabilitation, imaging, systems of care and outcomes. Evidence is presented for ischaemic stroke, ICH, cerebral venous thrombosis (CVT) and central retinal artery occlusion (CRAO). Evidence was identified through structured scanning of major peer-reviewed stroke, neurology and internal medicine journals (including The New England Journal of Medicine, JAMA, The Lancet, The Lancet Neurology, Stroke, European Stroke Journal, International Journal of Stroke, Neurology, Annals of Neurology, JAMA Neurology, Journal of Neurology, Neurosurgery and Psychiatry) and conference materials (including abstract proceedings) from major international stroke conferences during 2025 (ESOC, ISC, WSC), supplemented by within-board suggestions for high-impact or practice-relevant studies. Studies were first shortlisted by domain leads before review by the ESO Guideline Board. Candidate studies were discussed within the ESO Guideline Board, with inclusion based on their methodological quality, clinical relevance and potential impact across different healthcare settings. Disagreements were resolved by group discussion to achieve consensus. Conference-only results and trial protocols are explicitly described as preliminary/ongoing where applicable.

By highlighting practice-relevant findings, identifying key limitations and outlining research priorities, the ESO Guideline Board aims to provide the stroke community with rapid access to critical appraisal of emerging data while awaiting formal incorporation into evidence-based guidelines.

## Diagnostics and imaging

In 2025, key updates in stroke imaging focused on optimising imaging pathways for acute treatment decision-making and enhancing early aetiological work-up, emerging CT technologies and initiatives to improve methodological and reporting standards. The PRACTISE trial investigated IVT-eligible patients within 4.5 h using non-contrast CT only vs non-contrast CT/CT angiography/CT perfusion.^1^ Among 271 patients recruited between 2015 and 2018, significantly fewer patients received treatment in the multiparametric CT group compared with non-contrast CT alone (49.1% vs 67.6%), though time metrics and outcomes did not differ between groups.^1^ Whether reduced treatment rates reflect deselection of otherwise eligible patients or appropriate exclusion of stroke mimics remains uncertain, as PRACTISE was not powered to test non-inferiority in outcomes. The results remain inconclusive regarding impact on clinical practice.

In the DAYLIGHT prospective, randomised, open-label, blinded end-point trial, extending head-to-neck CT angiography caudally to include the upper thorax significantly increased by more than 5-fold the detection of cardioaortic thrombus compared with standard CT angiography, without delaying CT angiography completion.^2^

The primary technological innovation was photon-counting CT (pcCT), which offers superior dose efficiency, improved spatial resolution and intrinsic spectral sensitivity.^3^^,^^4^ Early applications suggest promise for differentiating contrast extravasation from haemorrhagic transformation, identifying carotid webs and assessing collateral circulation. However, widespread clinical implementation requires further validation.

A meta-analysis of 20 studies involving 14,599 patients established that intracranial arterial calcification (ICAC) detected on routine imaging is independently associated with significantly increased ischaemic stroke risk and showed a trend towards increased mortality.^5^ This finding suggests that ICAC may serve as an accessible imaging biomarker for stroke risk stratification.

A comprehensive review identified areas for methodological standardisation in cerebrovascular imaging research, noting that variability in the literature relates more to methodological heterogeneity than hardware limitations, particularly regarding imaging definitions for ischaemic core volumes, perfusion thresholds, metrics and use of independent core laboratories.^6^ The BRAINS guideline (Benchmarking Reporting Approach for Imaging in Neurological Studies) proposes key components including detailed imaging acquisition protocols, independent core laboratories for adjudication, imaging reader details with interrater reliability and validation processes, standardised reporting of extended TICI scores and reperfusion measures and full transparency in data analysis methods including software and thresholds used.^6^

### Potential practice implications

- The clinical utility of multiparametric CT vs non-contrast CT alone for treatment decision-making in IVT-eligible acute ischaemic stroke patients presenting within 4.5 h remains uncertain and requires further evidence.
- Extended CT angiography may increase detection of cardioaortic thrombus during acute code stroke imaging without delaying scan completion.
- Photon-counting CT is a promising technology for neurovascular imaging with potential applications in stroke, though its role in clinical practice awaits validation.
- Intracranial arterial calcification represents an accessible imaging biomarker that may inform stroke risk stratification.
- Standardised reporting frameworks such as the BRAINS guideline can improve quality and comparability of cerebrovascular imaging research.

### Key limitations and gaps

Major uncertainties remain regarding whether advanced imaging selection algorithms truly increase safety or efficacy of acute reperfusion therapies compared to simpler neuroimaging approaches. The real-world role of photon-counting CT in routine stroke care requires validation through adequately powered multicentre studies with patient-centred outcomes. In addition, its generalisability may be limited by substantial global variation in scanner availability, costs, technical requirements and expertise, particularly outside high-resource settings.

## Treatment of acute ischaemic stroke

Three independent RCTs established that EVT provides no benefit over best medical therapy (BMT) for MeVOs. DISTAL and ESCAPE-MeVO demonstrated that patients randomised to EVT experienced numerically higher rates of mortality, sICH and serious adverse events compared to BMT, which included intravenous thrombolysis (IVT) when eligible.^7^^,^^8^ DISCOUNT (NCT05030142) was stopped prematurely after interim analysis and full results have not been published (conference presentation; preliminary).^9^ A meta-analysis of the available data confirmed a lack of functional benefit with increased haemorrhagic complications and mortality.^10^

Five trials investigating intra-arterial (IA) thrombolysis after successful recanalisation yielded divergent results. PEARL and ANGEL-TNK demonstrated improved rates of excellent functional outcomes (mRS scores of 0–1) with IA alteplase (0.225 mg/kg) and tenecteplase (0.125 mg/kg), respectively, in anterior circulation LVO,^11^^,^^12^ though ANGEL-TNK showed no benefit in pre-specified secondary efficacy analyses and excluded patients receiving IVT.^12^ DATE evaluated the safety of different doses of IA tenecteplase given after successful EVT for an anterior circulation LVO and only doses of 0.0313 and 0.0625 mg/kg met the prespecified threshold on the risk of sICH.^13^ ATTENTION-IA reported increased sICH risk without functional benefit using 0.0625 mg/kg tenecteplase in posterior circulation.^14^ The prematurely terminated IA-TOP trial (NCT05897554) also reported no benefit for a higher tenecteplase dose (0.225 mg/kg) when given intra-arterially following successful EVT for posterior circulation LVO (conference presentation; preliminary).^15^ A meta-analysis of randomised data suggested that IA thrombolysis improves excellent functional outcomes without compromising safety in patients with LVO and successful recanalisation after EVT.^16^

The BRIDGE-TNK trial demonstrated superiority of intravenous tenecteplase before EVT compared with direct EVT, in patients eligible for IVT presenting to an EVT centre, with higher rates of good functional outcome (mRS scores of 0–2) and pre-procedural recanalisation.^17^ T-FLAVOR confirmed higher recanalisation rates prior to EVT with tenecteplase vs alteplase (0.6 mg/kg, as per the approved dose for stroke treatment in Japan) in LVO patients (conference presentation; preliminary).^18^

A prespecified secondary analysis of the IRIS individual patient data meta-analysis, encompassing 6 trials comparing bridging therapy with EVT alone in patients with LVO presenting directly at centres capable of endovascular treatment, revealed no association between IVT addition and increased risk of bleeding or modification of functional outcomes in patients with carotid tandem lesions. These findings suggest that tandem lesions should not preclude bridging therapy.^19^

The benefit of IVT beyond 4.5 h from stroke symptoms onset is still being explored. HOPE showed that alteplase administered 4.5–24 h after onset in patients who were not scheduled for EVT and had salvageable anterior circulation tissue on perfusion imaging with ischaemic core volume ≤ 70 mL increased the rate of excellent functional outcome, albeit with increased sICH.^20^ TRACE-5 reported higher rates of excellent functional outcome and recanalisation in patients treated with tenecteplase within 24 h for acute ischaemic stroke due to basilar artery occlusion, compared with BMT (conference presentation; preliminary).^21^ Patients with extensive ischaemic changes, as defined in the study protocol, in baseline imaging, were excluded from the trial. A 2025 meta-analysis of RCTs reported that tenecteplase (0.25 mg/kg) administered within 4.5–24 h (ROSE-TNK, TRACE-III, CHABLIS-T II, TIMELESS) improved excellent functional outcomes and recanalisation without increasing symptomatic ICH or 90-day mortality. However, these extended-window TNK trials enrolled highly selected populations, mostly using advanced imaging (CT/MR perfusion or DWI–FLAIR mismatch) and often requiring LVO confirmation.

### Potential practice implications

- Current evidence does not support routine EVT for patients presenting with acute ischaemic stroke and MeVO.
- More evidence is needed on the safety and efficacy of IA thrombolysis after successful EVT; enrolment into ongoing RCTs is encouraged.
- Evidence supports a benefit of intravenous tenecteplase prior to EVT (as bridging therapy) in IVT-eligible acute LVO patients presenting within 4.5 h from symptoms onset at EVT-capable centres.
- Current evidence does not support tandem lesions as a contraindication to bridging therapy
- Evidence suggests that late-window IV thrombolysis (4.5–24 h) with TNK may be considered in carefully selected patients meeting tissue-based advanced imaging criteria (perfusion or MRI mismatch) and with limited established infarction.

### Key limitations and gaps

Although all 3 MeVO trials were well-conducted and their results converge,^7-9^ there is a lingering question on the actual representativeness of trial participants when compared to the “real-world” patient population presenting with MeVOs. Pooled analyses from MeVO trials are needed to identify whether specific subgroups might benefit from EVT and to define the target population for potential follow-up trials.

The conflicting results from IA thrombolysis trials likely reflect disparities in study populations, circulation territories (anterior vs posterior), thrombolytic agents and doses used. Outcomes from ongoing trials (eg, TECNO, NCT05499832; 2BE3, NCT06034847; RESCUE-TNK, NCT05657470) are awaited to evaluate the utility of IA thrombolysis when given after successful EVT and the potential of this treatment intervention to be implemented across diverse clinical populations.

The utility of IVT after 4.5 h from symptoms onset relies on the availability of perfusion imaging and access to prompt EVT treatment, which varies considerably across healthcare systems.

## Treatment of acute ICH

The recently published ESO and European Association of Neurosurgical Societies (EANS) guideline provides an updated clinical framework for spontaneous ICH management, spanning acute care through secondary prevention.^22^

In 2025, new evidence further informed early blood pressure (BP) management in ICH. An individual patient data meta-analysis pooling the 4 INTERACT trials supported the safety of intensive BP lowering and confirmed improved functional recovery, with the greatest benefit observed when treatment begins within 3 h from symptoms onset.^23^ The ICH ADAPT-2 trial provided complementary reassurance that intensive targets (<140 mmHg) do not increase ischaemic lesion risk compared with conservative targets (<180 mmHg).^24^

Regarding anticoagulation reversal in factor Xa inhibitor-associated ICH, the ESO/EANS guideline highlights ongoing uncertainty about the balance between clinical benefits and potential adverse effects of andexanet alfa.^22^ A systematic review and meta-analysis of randomised and observational studies showed improved haemostatic efficacy with andexanet alfa in factor Xa inhibitor-associated ICH compared with usual care, but without a significant improvement in functional outcome, underscoring the need for higher-certainty comparative data and patient-centred endpoints.^25^

For surgical management, the guideline supports minimally invasive evacuation of the haematoma within 24 h of onset for patients with spontaneous supratentorial lobar ICH, based on the analysis of 4 RCTs consistently favouring minimally invasive surgery, but largely driven by the ENRICH trial.^22^^,^^26^ However, the MIND trial found no significant difference in 30-day mortality or 180-day disability between minimally invasive endoscopic evacuation and medical management in supratentorial ICH, though this trial was prematurely terminated following positive ENRICH results, limiting statistical power.^27^ For decompressive craniectomy in severe deep haemorrhage, a post-hoc SWITCH trial analysis demonstrated consistent treatment effect across locations (basal ganglia, internal capsule, thalamus), showing similar risk reductions in death or profound disability (mRS scores of 5–6 at 180 days) and mitigating concerns about topographic variability.^28^ A Cochrane review of surgery for spontaneous supratentorial ICH that included 24 RCTs with 4597 participants reported findings broadly consistent with those of the ESO/EANS guideline, while emphasising the need for further large, high-quality RCTs to better inform clinical practice.^29^

### Potential practice implications

- Evidence supports early, intensive BP lowering as a time-sensitive strategy in acute ICH, with greatest benefit when initiated within 3 h from symptoms onset.
- Andexanet alfa may be considered in selected patients with factor Xa inhibitor-related ICH after weighing the potential thrombotic risk, although clinical outcome benefit remains uncertain and availability varies.
- Minimally invasive surgical evacuation may be considered for lobar ICH and evidence suggests risk reduction in death or profound disability (mRS 5–6 at 180 days) with decompressive craniectomy for severe deep spontaneous ICH (Glasgow Coma Scale 8–13, National Institutes of Health Stroke Scale 10–30 and stable ICH volume 30–100 mL).

### Key limitations and gaps

The quality of evidence supporting intensive BP targets remains low due to heterogeneity in trial design, treatment protocols and intervention timing. Several ongoing trials (TIME-ICH NCT06760078, CLUTCH NCT06402968, Safety Study of Urapidil Alone or With Esmolol in Treating Acute Hypertensive Intracerebral Haemorrhage NCT06635707, MAX-ICH Pilot NCT06648369, I-CATCHER NCT06429332) aim to better define optimal early medical management strategies in patients with acute ICH. Comparative data between andexanet alfa and prothrombin complex concentrates remain limited.

Surgical management continues as an area of active investigation with low-quality evidence. Several ongoing trials evaluating minimally invasive surgery (EMINENT-ICH NCT05681988, EVACUATE NCT04434807, DIST NCT05460793) are expected to further inform patient selection and procedural timing in surgical candidates.

## Secondary stroke prevention

The TRIDENT trial demonstrated that triple-pill combination therapy (telmisartan 20 mg + amlodipine 2.5 mg + indapamide 1.25 mg) significantly reduced recurrent stroke rates compared with standard care after ICH, achieving a mean systolic BP of 127 mmHg without increase in serious adverse events (conference presentation; preliminary).^30^ This provides compelling evidence for intensive BP control aligned with the 2025 ESO/EANS ICH guideline target of ≤ 130/80 mmHg,^22^ while establishing polypill therapy with low-dose combination as a promising approach. The ESPRIT trial additionally showed that for hypertensive patients at high cardiovascular risk including those with stroke history, an even lower target (<120 mmHg vs < 140 mmHg) reduced major cardiovascular events with minor excess risk.^31^

CONVINCE represented the first dedicated trial assessing colchicine in non-cardioembolic stroke, showing no statistically significant reduction in major cardiovascular events (MACEs) overall.^32^ However, a pre-specified analysis identified a 38% reduction in recurrent MACE among patients with early C-reactive protein (CRP) response, suggesting potential for biomarker-guided patient selection.^33^ A study-level meta-analysis of 6 trials including 14,934 patients with prior stroke or coronary disease demonstrated that colchicine significantly reduced ischaemic stroke risk by 27% and MACE by 27%.^34^

Updated study-level meta-analysis of all 4 RCTs enrolling patients with embolic stroke of undetermined source (ESUS) confirmed no benefit for anticoagulation over antiplatelet therapy, with no differences by age, sex or atrial cardiopathy biomarkers,^35-37^ reinforcing 2022 ESO guideline recommendations.^38^

For large artery atherosclerosis, the ECST-2 2-year interim analysis showed no evidence for revascularisation benefit beyond optimised medical therapy in patients with asymptomatic or symptomatic carotid stenosis ≥ 50% at low or intermediate predicted stroke risk.^39^ The pilot CATIS-ICAD study showed numerical trends towards reduced recurrent ischaemic stroke with low-dose rivaroxaban plus aspirin vs aspirin alone in intracranial atherosclerotic disease.^40^

The OCEANIC-STROKE trial showed that factor XIa inhibition with asundexian significantly reduced ischaemic stroke risk without increasing major bleeding when added to standard antiplatelet therapy in patients with non-cardioembolic stroke or high-risk TIA (press release; preliminary).^41^ In contrast, in atrial fibrillation (AF), the phase 3 OCEANIC-AF trial comparing asundexian with apixaban was stopped early after asundexian was associated with a higher risk of stroke or systemic embolism, despite fewer major bleeding events.^42^

For AF, the CATALYST collaboration, an individual participant data meta-analysis of the 4 previous RCTs (TIMING, ELAN, OPTIMAS, START) that included 5441 participants, confirmed that early direct oral anticoagulant (DOAC) initiation within 4 days reduced recurrent ischaemic stroke without increasing the risk of sICH.^43^ The ATIS-NVAF trial demonstrated no net clinical benefit from adding antiplatelet therapy to anticoagulation in patients with nonvalvular AF and atherosclerotic cardiovascular disease, with increased bleeding risk,^44^ consistent with cardiology trials supporting anticoagulation monotherapy in stable coronary disease with AF.^45^ ARTESiA trial subgroup analysis confirmed that apixaban led to 7% absolute risk reduction in stroke or systemic embolism over 3.5 years in patients with device-detected subclinical AF and prior stroke or TIA, with a 3% absolute increase in major bleeding.^46^ The STABLED trial randomised 249 patients with non-valvular AF and prior ischaemic stroke to catheter ablation plus edoxaban vs edoxaban alone. Over a median follow-up of more than 3 years, the primary composite endpoint (recurrent ischaemic stroke, systemic embolism, all-cause death and hospitalisation for heart failure) occurred at similar rates in both groups (5.6% vs 4.9% per patient-year) (conference presentation; preliminary).^47^

A post-hoc ARCADIA analysis compared apixaban (2.5–5 mg twice daily) with aspirin (81 mg) in cryptogenic stroke patients with cancer history (*n* = 137). The primary outcome (composite of major ischaemic or major haemorrhagic events) did not differ significantly between groups (13.1% apixaban vs 21.2% aspirin).^48^

A matched cohort study using Medicare fee-for-service data from 2016 to 2022 (device arm *n* = 1132; control arm *n* = 4376) reported significantly lower recurrent ischaemic stroke risk for patent foramen ovale closure in patients over 60 years.^49^ However, 30-day mortality rates did not differ, and venous thromboembolism and AF or flutter were significantly more common in the device arm.

### Potential practice implications

- In patients with prior ICH, BP control to ≤ 130/80 mmHg as recommended by the 2025 ESO/EANS guideline is further supported by the TRIDENT trial; low dose polypill combination therapy represents a proven strategy to achieve this target.
- In patients with ESUS, antiplatelet therapy remains first-line treatment as recommended by the 2022 ESO guideline, further confirmed by updated meta-analysis of 4 RCTs; routine anticoagulation is not recommended.
- In patients with AF-related stroke, evidence supports early DOAC initiation to reduce recurrent ischaemic events; adding antiplatelet therapy provides no additional benefit and increases bleeding risk in those with concomitant atherosclerotic cardiovascular disease. The role of rhythm control in secondary stroke prevention remains uncertain.
- In non-cardioembolic ischaemic stroke or high-risk TIA, factor XIa inhibition with asundexian represents a promising novel treatment strategy for reducing recurrent ischaemic stroke without excess bleeding.

### Key limitations and gaps

Optimal BP target for secondary prevention after ischaemic stroke remains to be established. There are several planned or recruiting RCTs comparing more intensive (<120 mmHg) vs standard (<140 mmHg) targets, which should provide some clarity in the near future (OPTIMAL Stroke NCT04036409; EPICS-Pilot NCT04647292).

Better patient selection is needed to inform the choice of anticoagulation over antiplatelet treatment in patients without known AF. Results from the MOSES trial (NCT03961334) are anticipated to inform this issue. Subgroup analyses of OCEANIC-STROKE will provide further insights on the role of dual pathway inhibition for secondary stroke prevention in patients with ESUS. The ongoing LIBREXIA-STROKE trial, evaluating milvexian (another factor XIa inhibitor), will add to the evidence base for this novel therapeutic class.

For patients with known AF, a new frontier is to determine the best treatment option for patients with stroke despite optimal anticoagulation. The ELAPSE (NCT05976685) and INTERCEPT (NCT05723926) trials should help inform future strategies in patients with stroke and AF who remain at elevated risk of stroke recurrence despite ongoing anticoagulation. The ongoing EAST-STROKE trial (NCT05293080) is evaluating early rhythm control therapy, including catheter ablation, in acute ischaemic stroke patients with AF to determine whether this strategy reduces cardiovascular events. The CLOSE-2 trial (NCT05387954) may provide further evidence on patent foramen ovale closure in stroke patients aged 60–80 years.

Finally, the potential benefit of anti-inflammatory treatment in secondary stroke prevention remains unproven and CASPER (ACTRN12621001408875) will investigate whether high sensitivity CRP might be helpful to guide better patient selection for colchicine treatment. The ongoing CoVasc-ICH 2 (NCT06587737) trial additionally aims to determine the acute and long-term effects of reducing thrombo-inflammation with colchicine in patients with acute ICH.

## Stroke in the young and less common causes

Large international observational studies have provided further insight into traditional and non-traditional risk factors for young onset ischaemic stroke, which is particularly important as incidence increases globally, driven mainly by cryptogenic stroke. A Mendelian randomisation study from the Stroke Genetic Network and Early-Onset Stroke Consortium including almost 7000 young cases reported associations among higher body mass index, BP, type 2 diabetes and lower high-density lipoprotein cholesterol with young-onset stroke.^50^ The Global Burden of Disease Study 2021 systematic analysis highlighted the role of high systolic BP, ambient particulate matter pollution, high body mass index, high low-density lipoprotein cholesterol and high fasting blood glucose in increased stroke-related disability-adjusted life-years among young adults from 1990 to 2021.^51^ The multicentre SECRETO study reported that traditional risk factors significantly contribute to young-onset cryptogenic ischaemic stroke without patent foramen ovale, whereas non-traditional factors, most notably migraine with aura particularly in women, appear more critical for cryptogenic stroke with patent foramen ovale.^52^ Another SECRETO sub-study highlighted the association between self-perceived stress and young-onset cryptogenic stroke in women but not men, even after robust adjustment for cardiovascular risk factors.^53^

The international prospective observational DOAC-CVT study enrolled 619 patients with CVT between 2021 and 2024, demonstrating that during a 6-month follow-up, rates of recurrent thrombosis and major bleeding did not differ between patients treated with DOAC vs vitamin K antagonists.^54^ The 12-month results of the EXCOA-CVT cluster RCT and prospective cohort study showed no significant difference in recurrent venous thromboembolism or major bleeding risk between short-term (3–6 months) and long-term (12 months) anticoagulation, in patients with CVT without indication for permanent anticoagulation (conference presentation; preliminary).^55^ The 24-month follow-up is ongoing.

For cervical artery dissection, the retrospective STOP-CAD study (over 4000 patients) showed no overall association between anticoagulation and ischaemic stroke recurrence at 6 months compared with antiplatelet therapy. However, a secondary analysis demonstrated that patients with occlusive dissection who received anticoagulation experienced lower recurrence risk compared to antiplatelet therapy.^56^ An individual patient data meta-analysis of CADISS and TREAT-CAD trials (*n* = 444) showed fewer primary endpoint events with anticoagulation vs antiplatelets (1.4% vs 4.4%), although the difference was not statistically significant.^57^ Additionally, during the extended follow-up period of TREAT-CAD, outcomes between 3 and 6 months after randomisation were rare, occurred at similar rates in both groups and were exclusively haemorrhagic events.^58^ In a registry study of females with prior cervical artery dissection, pregnancy was not associated with an increased overall long-term risk, though event rates were numerically higher during pregnancy through postpartum (3.17%/year) vs outside pregnancy (0.67%/year), with all recurrent dissections occurring in the 6-week postpartum period.^59^

### Potential practice implications

- Interventions addressing both traditional risk factors (obesity, hypertension, diabetes, dyslipidaemia) and non-traditional factors (migraine management, stress reduction) may be relevant for comprehensive prevention strategies in young-onset stroke.
- In CVT patients, direct oral anticoagulants may be considered a reasonable treatment option compared with vitamin K antagonists. More data are awaited on optimal treatment duration of anticoagulation in patients without indication for permanent treatment.
- In cervical artery dissection, both anticoagulants and antiplatelets can be considered; when balancing benefits and risks, it is reasonable to consider occlusive dissection status in individualised decision-making regarding a short course of anticoagulation. In women with prior cervical artery dissection, pregnancy does not increase overall long-term risk, though the risk may be elevated in the postpartum period.

### Key limitations and gaps

The main limitation in young-stroke studies is observational study design, leading to lower-quality evidence, more prone to bias, confounding and limited ability to establish causality. Studies relying on patient-reported exposures such as self-perceived pre-stroke stress warrant consideration of recall bias when interpreting results. The lower risk of recurrent stroke with anticoagulation in occlusive dissection subgroups is not derived from randomised comparisons, and caution should be exercised when interpreting these data. Beyond study design limitations, there remains limited evidence on which preventive interventions are most effective and feasible in young adults, across different regions and healthcare systems. Results from the ongoing Y-SCOPE study (NCT06820411) assessing optimal prevention strategies and outcome improvement in young ischaemic stroke patients are awaited. Future research should prioritise population-based cohort studies and larger, adequately powered RCTs to provide reliable data for guiding individual patient care and treatment decisions.

## Outcomes and prognostication

Two large meta-analyses demonstrate ongoing high residual recurrence risk in patients with ischaemic stroke or TIA. In a meta-analysis of 171,068 patients with minor stroke or TIA, the pooled incidence of recurrent stroke was 5.94 events per 100 person-years in the first year, with cumulative 5- and 10-year risks of 12.5% and 19.8%, respectively.^60^ In another meta-analysis, the cumulative incidence of recurrent ischaemic stroke after AF-related stroke approached 4% per year despite high rates of oral anticoagulation, with even higher rates in patients with stroke despite anticoagulation (7.2% per year).^61^

Post-stroke epilepsy (PSE) is associated with increased mortality and reduced quality of life. The ODYSSEY registry showed that the 5-year cumulative risk of PSE after ischaemic stroke and ICH in young adults was 3.7% and 7.6%, respectively, with most presenting seizures occurring in the first year.^62^ Cortical involvement and early post-stroke seizure (<7 days) were strongly associated with PSE, and the SELECT and CAVE scores performed well for risk stratification.^62^ The PROLEVIS trial compared early administration of levetiracetam with placebo in patients with cortical ischaemic stroke within 14 days; the primary outcome of first symptomatic seizure did not differ between groups (conference presentation; preliminary).^63^

In patients with ICH, the Edinburgh Risk Score incorporating APOE genotyping was associated with recurrent ICH during long-term follow-up.^64^ In patients with stroke due to carotid atherosclerosis, MRI imaging to detect intra-plaque haemorrhage improved risk stratification when incorporated into a risk score (IMPROVE), identifying patients at greatest risk of recurrent ischaemic stroke receiving best medical therapy.^65^

### Potential practice implications

- The long-term risk of recurrent stroke is considerable after minor stroke/TIA and AF-related stroke, highlighting persistent risk beyond the acute phase and reinforcing the need to optimise long-term goal-directed secondary prevention.
- Risk prediction scores might be used to counsel younger patients of their future risk of PSE. Routine administration of prophylactic anti-seizure medication after ischaemic stroke has not demonstrated benefit for seizure prevention.
- Carotid plaque imaging with high-resolution MRI may improve prognostication of future ischaemic stroke risk and could inform individualised treatment decisions.

### Key limitations and gaps

Future trials of prophylactic anti-seizure medication may inform whether selection of acute ischaemic stroke patients that may benefit from this therapeutic approach can be enriched by identifying high-risk patients using prediction scores. Future RCTs comparing revascularisation with medical therapy may also adopt selection strategies based on risk scores that incorporate MRI markers of plaque vulnerability. More broadly, key remaining gaps include determining which prediction tools and imaging biomarkers should change clinical decisions (including defining actionable thresholds and harmonising how these measures are reported and interpreted across centres) and how to integrate them into clinical pathways.

## Neurorehabilitation

Recent RCTs have evaluated novel neuromodulation and pharmacological approaches for post-stroke rehabilitation with mixed results. The EMAGINE trial, a double-blind, sham-controlled study conducted in the United States, evaluated electromagnetic network targeting field therapy paired with home-based exercise in subacute stroke patients with moderate-to-severe disability.^65^ The trial was stopped early after enrolling 100 participants due to futility in the primary endpoint of disability reduction at day 90 (mRS), though a promising trend emerged for functional independence (26% achieved mRS 0–1 in the active group vs 10% in sham group), with no device-related serious adverse events.^66^

The TRANSPORT2 phase 2 multicentre triple-blind RCT (*n* = 129) found that adding bi-hemispheric transcranial direct current stimulation at 2 or 4 mA to modified constraint-induced movement therapy in patients 1–6 months post-ischaemic stroke did not further reduce upper limb motor impairment compared with modified constraint-induced movement therapy plus sham, though transcranial direct current stimulation was safe, well-tolerated and feasible.^67^

ESTREL, a multicentre RCT with 610 participants, reported that adding levodopa/carbidopa to standardised rehabilitation therapy based on active task-oriented training did not significantly improve motor function at 3 months compared with placebo, with no difference in adverse events.^68^

A small trial (*n* = 43) comparing home-based self-managed rehabilitation programmes (STRONG online platform vs paper exercises) yielded modest motor improvements in both arms, yet STRONG participants reported superior arm/hand use quality and increased self-efficacy, suggesting that accessible home-based delivery can improve functional outcomes.^69^ A network meta-analysis of 24 trials including 868 stroke patients suggested that smart rehabilitation therapy combined with transcranial magnetic stimulation had highest effectiveness in improving activities of daily living.^70^ The ESO guideline on motor rehabilitation, published in 2025, recommends at least 20 h of additional repetitive upper limb and gait practice, high-intensity walking training in chronic stroke patients with stable cardiovascular status and consideration of behavioural transfer packages for upper limb task-specific training.^71^

The SEARCH trial (*n* = 158) found that structured visual scanning training for hemianopia showed no superiority over sham training in the primary outcome (visual function questionnaire NEI VFQ-25) from baseline to 26 weeks, yet both groups improved substantially, suggesting robust placebo/expectancy effects warranting further mechanistic research.^72^

### Potential practice implications

- Current trial evidence does not support routine use of levodopa to enhance post-stroke motor recovery.
- In the studied population, bi-hemispheric transcranial direct current stimulation at standard doses was not associated with improved outcomes when added to intensive upper limb rehabilitation.
- Electromagnetic network targeting field therapy appears safe with promising trends towards improved functional independence; however, larger definitive trials are needed before clinical implementation.
- Both digital and paper-based home rehabilitation programmes can produce functional gains; online platforms may offer additional benefits for self-efficacy.
- Providing at least 20 h of additional repetitive upper limb and gait practice is consistent with current ESO motor rehabilitation guideline recommendations, with high-intensity gait training strongly recommended for chronic stroke patients with stable cardiovascular status.

### Key limitations and gaps

Several trials evaluating novel neuromodulation approaches were underpowered or prematurely terminated, limiting the ability to draw definitive conclusions. Protocols for optimising dose, combination and delivery of advanced neuromodulation therapies are still being established. Long-term real-world effectiveness and adherence data, particularly for home-based and digital interventions, are needed to guide clinical implementation.

## Post-stroke cognitive impairment and dementia

Recent evidence in this domain includes identification of risk factors, diagnostic frameworks and therapeutic interventions for post-stroke cognitive impairment (PSCI) and dementia (PSD). The large prospective DEMDAS study investigated risk factors for PSCI and PSD in dementia-free stroke patients followed over 5 years.^73^ The cumulative incidence of PSD was 3.1% at 6 months, rising to 8.8% at 5 years. Key risk factors for PSD included greater index stroke severity, older age, lower educational attainment, acute-phase cognitive impairment, lower functional status, AF, metabolic syndrome, stroke recurrence and neuroimaging markers of cerebral small-vessel disease. Metabolic syndrome was strongly associated with late-onset PSD (>6 months), whereas factors related to index stroke severity were more strongly related to early-onset PSD (≤6 months). Patients who received acute reperfusion therapies had a 65% lower risk of PSD.^73^ The prospective IDEA3 study demonstrated that stroke patients with positive baseline amyloid PET scans had substantially higher risk of PSD, emphasising the role of co-pathology.^74^ Patients with diffusion-weighted imaging-negative TIA exhibited a rate of cognitive decline comparable to that of stroke patients after controlling for vascular risk factors.^75^

The newly revised VasCog-2–WSO diagnostic criteria for vascular dementia adopt a spectrum-based framework using the term vascular cognitive impairment and dementia (VCID).^76^ Major revisions include requirement that neurocognitive disorder persists for > 3 months, clear temporal relationship between cerebrovascular disease and cognitive decline, integration of fluid and neuroimaging biomarkers, distinction between possible and probable VCID and introduction of a preclinical “at-risk VCID” category.^76^

Regarding therapeutic interventions, a meta-analysis of 19 studies (*n* = 875) found that computerised cognitive training might enhance general cognitive function in patients with PSCI.^77^ Another systematic review reported that brain stimulation therapies, predominantly transcranial direct current stimulation, were associated with improved post-stroke cognitive outcomes.^78^ An RCT of cardiorespiratory training starting 2 months post-stroke demonstrated better executive and global cognitive function at 12 months, though it did not influence the primary imaging outcome.^79^ A further trial in chronic stroke survivors with executive dysfunction found that strategy training improved immediate societal participation, though the effect did not persist at 3 months.^80^

### Potential practice implications

- Diagnostic criteria for vascular dementia were substantially revised.
- Metabolic syndrome is associated with late-onset PSD, suggesting effective treatment of metabolic syndrome in stroke survivors as a potential prevention target.
- DWI-negative TIA is associated with cognitive decline after the index event, supporting closer attention to cognitive outcomes and follow-up strategies in this group.
- Positive amyloid PET status is associated with higher risk of PSCI.
- Promising novel therapeutic strategies for PSCI include brain stimulation and computerised cognitive training.
- Cardiorespiratory training after stroke is safe and may improve executive and global cognitive function.

### Key limitations and gaps

Multiple studies investigating PSCI demonstrate high heterogeneity in populations, interventions and outcome measures, necessitating improved standardisation and adequately powered RCTs. Long-term effects of brain stimulation and computerised cognitive training remain unclear. Larger trials are needed to investigate cardiorespiratory training efficacy and define optimal training regimens. Cognitive endpoints should be incorporated into stroke trials given the high prevalence and functional impact of PSCI. The ongoing DISCOVERY trial (NCT04916210) will enrol 8000 dementia-free stroke survivors and aims to elucidate mechanisms of brain resilience and susceptibility to PSCI using clinical, imaging and biomarker data.

## Systems of care and implementation

Recent evidence focused on prehospital delivery models and implementation strategies to shorten time-to-treatment, including mobile stroke units (MSUs) and workflow optimisation for EVT, and adherence to key acute care targets through care bundles. Analysis of the BEST-MSU trial, a multi-site study in 7 US cities, demonstrated that MSU deployment was economically favourable from a Medicare perspective, with lifetime incremental cost-effectiveness ratios of US$94,710 per quality-adjusted life year (QALY) overall for IVT-eligible patients and US$31,259/QALY in patients without pre-existing disability.^81^ In a separate retrospective observational cohort study using data from 106 US hospitals in the Get With The Guidelines-Stroke registry, prehospital management with MSU was associated with lower global disability at discharge compared with standard emergency medical services in patients potentially eligible for IVT.^82^ The Australian MSU-TELEMED programme demonstrated that tele-neurology staffing can be safe, productive and potentially cost-saving, supporting hybrid deployment models (conference presentation; preliminary).^33^

The OPTIMAL cluster trial tested a behaviour-change package (PEITER: Persuasion, Enablement, Incentivisation, Training, Education, and Review) across 16 hospitals treating 1288 LVO patients with EVT. The intervention was associated with shorter door-to-puncture times and improved 90-day functional outcomes (mRS scores of 0–3) from 55.4% to 66.4% (conference presentation; preliminary).^83^

The MAP-STROKE study developed a personalised Bayesian prehospital triage algorithm for suspected LVO patients, with modelling suggesting a 2.1% absolute increase in mRS scores of 0–2 by optimising bypass decisions to comprehensive stroke centres (conference presentation; preliminary).^33^

A population-based study of 1821 ICH patients found that achieving early care bundle targets (BP, glucose, oxygen, temperature control) and shorter onset-to-admission times were associated with improved 3-month functional outcomes (mRS scores of 0–3).^84^

### Potential practice implications

- Mobile stroke unit deployment may improve functional outcomes and appear cost-effective in high-volume systems, though evidence is primarily from urban settings in the United States and Australia.
- Workflow behaviour-change packages can reduce treatment delays and were associated with improved outcomes for EVT in a cluster trial setting.
- Personalised prehospital triage algorithms show promise for optimising LVO patient routing.
- Early care bundle implementation for ICH was associated with better functional outcomes in a population-based study.

### Key limitations and gaps

Mobile stroke unit evidence is primarily from high-resource and urban settings and cost-effectiveness may differ by geography and service volume. Modelling-based prehospital triage tools (eg, MAP-STROKE) require prospective validation, including assessment of potential harms (eg, overtriage, inappropriate bypass and capacity constraints). The ICH care bundle study was observational, limiting causal inference. Ongoing pre-hospital trials include FASTEST (recombinant factor VIIa in ultra-early ICH) and EAST (prehospital ICH interventions).

## Other topics

Concerning neuroprotection, a post-hoc meta-analysis of individual patient data pooling ESCAPE-NA1, ESCAPE-NEXT and FRONTIER RCTs suggested a potential functional benefit of nerinetide in patients treated within 3 h and selected for reperfusion (56% vs 48% responders at 90 days; adjusted OR 1.48; 95% CI, 1.07–2.06; *P* = .017), without an apparent safety signal.^85^ These findings are hypothesis-generating and warrant confirmation in a dedicated phase 3 RCT.

Two RCTs evaluated thrombolysis in acute CRAO within 4.5 h. The THEIA trial (*n* = 70) compared intravenous alteplase (0.9 mg/kg) with oral aspirin (300 mg) and found no significant difference in visual acuity improvement of at least 0.3 LogMAR at 1 month (66% vs 48%; *P* = .95). Safety was acceptable, with 1 asymptomatic ICH in the alteplase group and no symptomatic haemorrhages.^86^ The TenCRAOS trial (*n* = 78) compared intravenous tenecteplase (0.25 mg/kg) with aspirin and also showed no benefit for the primary outcome (visual acuity ≤ 0.7 LogMAR at 30 days: 20.0% vs 23.7%, *P* = .69). However, adverse events occurred more frequently in the tenecteplase group, including one fatal ICH (conference presentation; preliminary).^87^

The CLOSURE-AF trial (*n* = 912) evaluated left atrial appendage occlusion (LAAO) vs physician-directed medical care in primary prevention, in patients with AF at high risk of both stroke (mean CHA₂DS₂-VASc 5.2) and bleeding (mean HAS-BLED 3.0). Over a median follow-up of 3 years, LAAO failed to achieve noninferiority for the composite of stroke, systemic embolism, cardiovascular or unexplained death or major bleeding (16.83 vs 13.27 per 100 person-years; adjusted HR 1.28, 95% CI, 1.01–1.62) (conference presentation; preliminary).^88^

The CREST-2 trial evaluated intensive medical therapy vs carotid revascularisation for asymptomatic high-grade stenosis. In the stenting trial (*n* = 1245), carotid artery stenting significantly reduced the 4-year composite endpoint of stroke or death compared with medical therapy alone (2.8% vs 6.0%, *P* = .02). In the endarterectomy trial (*n* = 1240), carotid endarterectomy did not achieve statistical significance (3.7% vs 5.3%, *P* = .24). These findings suggest that while intensive medical therapy alone yields low stroke rates, the addition of stenting led to a lower risk of a composite of perioperative stroke or death or ipsilateral stroke within 4 years, compared with medical management alone. Carotid endarterectomy did not lead to a significant benefit.^89^

### Potential practice implications

- Recent randomised trials (THEIA, TenCRAOS) do not support routine use of intravenous thrombolysis for acute CRAO; enrolment into ongoing RCTs is encouraged.
- In high-risk AF patients in a primary prevention setting, LAAO failed to demonstrate benefit over best medical therapy in CLOSURE-AF; its role in different risk populations continues to be evaluated in ongoing trials.
- Carotid artery stenting on top of intensive medical management may provide additional benefit in selected patients with asymptomatic high-grade carotid stenosis.

### Key limitations and gaps

Both THEIA and TenCRAOS were likely underpowered and enrolled few patients within 3 h of onset. The larger REVISION trial (NCT04965038), including biomarkers, is ongoing, and results are awaited. Individual patient-level meta-analyses across these trials may further inform whether earlier treatment benefits specific patient subgroups.

Following the neutral CLOSURE-AF results, ongoing trials are evaluating left atrial appendage occlusion in different risk populations (CHAMPION-AF, NCT04394546; CATALYST, NCT04226547; LAAOS-4, NCT05963698). For asymptomatic carotid stenosis, the benefit of stenting was based on a small number of events, results from high-volume certified operators may not reflect broader practice, and evolving medical therapies may further reduce the benefit of revascularisation.

## Discussion

This first ESO Annual Stroke Evidence Update summarises major developments in 10 key domains of stroke care in 2025 (Table 1). Key findings include 3 RCTs showing no benefit of routine EVT over best medical therapy for stroke due to MeVO, refinement of BP targets for ICH with evidence supporting intensive control, validation of polypill strategies for secondary prevention after ICH, and promising data on asundexian, a novel factor XIa inhibitor, for non-cardioembolic stroke (Table 2). Technological innovations in neuroimaging, particularly photon-counting CT, provide new possibilities for stroke diagnosis and risk assessment, while advances in stroke systems of care, from MSUs and algorithm-based prehospital triage to workflow optimisation and early care bundles, have further demonstrated meaningful improvements in patient outcomes.

**Table 1:** Core findings by domain.

**Domain**	**Key findings**
**Diagnostics and imaging**	**Multiparametric CT in IVT-eligible patients:** Fewer treatments (49.1% vs 67.6%) with similar outcomes (PRACTISE, 271 patients, 2015–2018) **Extended CTA including the heart**: Higher cardioaortic thrombus detection (8.8% vs 1.7%) without delaying CTA completion (DAYLIGHT, 465 patients) **Photon-counting CT:** Superior dose efficiency, resolution, spectral sensitivity; promising applications; requires validation **Intracranial arterial calcification:** Independently associated with increased stroke risk and mortality trend **BRAINS guideline:** Standardised reporting framework for imaging research
**Acute ischaemic stroke**	**EVT for MeVO:** No benefit vs medical therapy; higher rates of symptomatic ICH (DISTAL, ESCAPE-MeVO, DISCOUNT) **IA thrombolysis post-successful EVT:** Improved excellent functional outcome in anterior circulation (PEARL, ANGEL-TNK); increased symptomatic ICH without benefit in posterior circulation (ATTENTION-IA, IAT-TOP) **IVT with tenecteplase before EVT:** Improved 90-day functional independence compared to direct EVT (BRIDGE-TNK); higher recanalisation vs alteplase (T-FLAVOR) **Bridging Therapy** (IVT and EVT): Tandem carotid occlusions can be safely treated **Extended-window IVT:** IV alteplase 4.5 to 24 h in patients with salvageable brain tissue and ischaemic core ≤ 70 mL increased excellent functional outcome (mRS 0–1) but increased symptomatic ICH in anterior circulation (HOPE); IV tenecteplase within 24 h for basilar occlusion without extensive baseline ischaemic changes improved functional outcomes without safety concerns (TRACE-5)
**Acute ICH**	**ESO/EANS guideline:** Comprehensive evidence-based recommendations published **Intensive BP lowering:** Safe and improves functional outcomes; greatest benefit within 3 h (IPD meta-analysis 4 INTERACT trials); no increased ischaemic lesion risk with target < 140 mmHg (ICH ADAPT-2) **Andexanet alfa:** Improved haemostatic efficacy but no significant difference in functional outcome (meta-analysis) **Minimally invasive evacuation for lobar ICH:** Supported by guideline (4 RCTs, mostly driven by ENRICH); MIND showed no significant difference (prematurely terminated) **Decompressive craniectomy**: Consistent benefit across deep ICH locations (post-hoc SWITCH); Cochrane review (24 RCTs, 4597 patients) consistent with ESO/EANS guideline
**Secondary stroke prevention**	**BP control post-ICH:** Polypill (TRIDENT) achieved mean SBP 127 mmHg, reduced recurrent stroke (TRIDENT); target < 120 mmHg reduced MACE vs < 140 mmHg (ESPRIT) **Colchicine:** No significant MACE reduction overall; 38% reduction in patients with early CRP response (CONVINCE); meta-analysis of ischaemic stroke and coronary artery disease patients showed 27% reduction in stroke and MACE (6 RCTs, 14,934 patients) **Revascularisation in carotid stenosis**: No benefit beyond medical therapy in low/intermediate risk (ECST-2); rivaroxaban + aspirin in ICAD showed numerical trends of benefit (pilot CATIS-ICAD) **Anticoagulation in ESUS:** No benefit vs antiplatelet (updated meta-analysis 4 RCTs) **Asundexian:** Reduced ischaemic stroke without increased bleeding in non-cardioembolic stroke/TIA (OCEANIC-STROKE) **DOAC in AF:** Early initiation in patients with ischaemic stroke reduced recurrent events within 30 days without increase in symptomatic ICH (CATALYST IPD meta-analysis of 4 RCTs); apixaban in device-detected subclinical AF: 7% absolute risk reduction in stroke, 3% increase in bleeding (ARTESiA subgroup) **AF and atherosclerosis:** Adding antiplatelet to DOAC increases bleeding without benefit (ATIS-NVAF) **Catheter ablation in AF:** Adding catheter ablation to edoxaban was not associated with benefit (STABLED) **PFO closure > 60 years:** Lower recurrent stroke; increased VTE and AF/flutter (Medicare observational data)
**Young stroke and less common causes**	**Risk factors:** Risk factors for young-onset ischaemic stroke include higher BMI, increased BP levels, diabetes and lower HDL (Mendelian randomisation study) **CVT:** DOACs vs VKAs no difference in recurrence/bleeding at 6 months (DOAC-CVT) **CeAD:** Anticoagulation vs antiplatelet no overall difference (STOP-CAD; CADISS + TREAT-CAD meta-analysis *n* = 444); lower recurrence in occlusive dissection with anticoagulation (STOP-CAD)
**Outcomes and prognostication**	**Recurrence risk:** 6%, 13% and 20% at 1, 5 and 10 years, respectively (meta-analysis of observational data) **AF stroke recurrence:** ~ 4% annually despite anticoagulation; ~ 7% annually in patients with previous stroke despite anticoagulation **Post-stroke epilepsy:** 3.7% (ischaemic) and 7.6% (ICH) 5-year cumulative risk in young adults; cortical involvement and early seizure (<7 days) strongest predictors (ODYSSEY) **Anti-seizure prophylaxis:** No benefit for levetiracetam (PROLEVIS) **Risk prediction:** Edinburgh Score + APOE associated with recurrent ICH; IMPROVE score identifies high-risk recurrent ischaemic stroke in carotid atherosclerosis
**Neuro-rehabilitation**	**Levodopa:** No motor improvement at 3 months (ESTREL) **Bi-hemispheric tDCS:** No additional benefit; safe and feasible (TRANSPORT2, phase 2) **Electromagnetic network targeting field therapy:** Stopped early for futility; promising trend excellent functional outcome (26% vs 10%); safe (EMAGINE, phase 2) **Smart rehabilitation + rTMS:** Highest ADL effectiveness (meta-analysis of 24 trials) **Visual scanning training**: No superiority vs sham; both improved (SEARCH) **ESO 2025 guideline on motor rehabilitation:** ≥ 20 h additional upper limb/gait practice; high-intensity gait training for chronic stroke with stable cardiovascular status
**Post-stroke cognitive impairment**	**Post-stroke dementia:** 3.1% at 6 months, 8.8% at 5 years; risk factors include stroke severity, age, education, cognitive impairment, AF, metabolic syndrome, recurrence, small-vessel disease; reperfusion therapy associated with 65% lower risk (DEMDAS, 5-year follow-up) **Amyloid co-pathology:** Positive PET is associated with higher risk of post-stroke cognitive impairment (IDEA3) **DWI-negative TIA:** Associated with cognitive decline after the index event **VasCog-2-WSO diagnostic criteria:** Vascular cognitive impairment and dementia spectrum approach, with refined cognitive domain definitions, integration of biomarkers and a new preclinical “at-risk” category **Interventions:** Computerised training (meta-analysis, 19 studies), tDCS and cardiorespiratory training show promise
**Systems of care**	**Mobile stroke units:** Economically favourable ICERs (BEST-MSU, 7 US cities); lower disability vs standard EMS (106 hospitals, GWTG-Stroke); tele-neurology safe and cost-saving (Australian MSU-TELEMED) **Workflow optimisation:** PEITER package increased 90-day favourable functional outcomes (OPTIMAL cluster trial) **Prehospital triage:** MAP-STROKE personalised Bayesian algorithm modelling suggests 2.1% absolute increase in good functional outcome **ICH care bundles:** Improved 3-month functional outcomes (population-based study)
**Other topics**	**Neuroprotection (nerinetide):** post-hoc individual patient data meta-analysis pooling ESCAPE-NA1, ESCAPE-NEXT and FRONTIER found an hypothesis-generating signal of improved 90-day functional response in reperfusion-selected patients treated within 3 h (aOR 1.48; 95% CI, 1.07–2.06) **Central retinal artery occlusion**: no benefit for thrombolysis vs aspirin within 4.5 h (both *P* > .05); 1 fatal ICH with Tenecteplase (THEIA, *n* = 70 and TenCRAOS trials, *n* = 78) **Left atrial appendage occlusion in patients with AF**: Failed noninferiority vs medical care in high-risk AF (CLOSURE-AF, *n* = 912) **Asymptomatic high-grade carotid stenosis**: Stenting added to intensive medical therapy reduced 4-year stroke/death, compared with BMT only; carotid endarterectomy did not lead to a significant benefit (CREST-2)

Abbreviations: ADL, activities of daily living; AF, atrial fibrillation; APOE, apolipoprotein E; BMI, body mass index; BMT, best medical therapy; BP, blood pressure; CeAD, cervical artery dissection; CVT, cerebral venous thrombosis; DOAC, direct oral anticoagulant; DWI, diffusion-weighted imaging; EMS, emergency medical services; ESUS, embolic stroke of undetermined source; GWTG, Get With The Guidelines; HDL, high-density lipoprotein; IA, intra-arterial; ICAD, intracranial atherosclerotic disease; ICER, incremental cost-effectiveness ratio; IPD, individual patient data; IVT, intravenous thrombolysis; MACE, major adverse cardiovascular events; MSU, mobile stroke unit; PET, positron emission tomography; PFO, patent foramen ovale; rTMS, repetitive transcranial magnetic stimulation; SBP, systolic blood pressure; tDCS, transcranial direct current stimulation; VKA, vitamin K antagonist; VTE, venous thromboembolism.

**Table 2:** Key messages for clinical practice by domain. Symbols summarise the overall direction of the message: ▲ indicates evidence suggesting potential benefit/utility or supportive signal; ● indicates neutral, uncertain or mixed evidence; ▼ indicates evidence suggesting harm, unfavourable signal or lack of clinical benefit.

**Domain**	**Key message for clinical practice**
**Diagnostics and imaging**	▲ Photon-counting CT is a promising technology for cerebrovascular diagnostics with improved image quality and reduced radiation exposure (OBS) ▲ Extended CTA including the heart increases detection of cardioaortic thrombus without delaying CTA completion (RCT) ▲ Intracranial arterial calcification is a significant risk factor for incident stroke (MA) ▲ Recognition and systematic addressing of methodological limitations in cerebrovascular imaging research represents a key step in evidence quality improvement (GL)
**Treatment of acute ischaemic stroke**	▼ Current evidence does not support routine EVT for acute MeVO. Optimal medical therapy, including IVT, when eligible, should be administered (RCT, MA) ▲ Patients with LVO who are eligible for thrombolytic therapy should receive TNK prior to EVT (RCT). Carotid tandem occlusion alone should not preclude bridging therapy (IPDMA). ▲ IVT may be considered between 4.5 and 24 h from symptom onset for selected patients (RCT, MA) • Upcoming RCT data will inform the utility of intra-arterial thrombolysis after successful EVT (RCT, MA)
**Treatment of acute ICH**	▲ Early intensive SBP reduction to < 140 mmHg improves outcomes after spontaneous ICH without increasing ischaemic brain injury (IPDMA, GL) • Andexanet alfa shows improved haemostatic efficacy in factor Xa inhibitor-associated ICH, but functional benefit remains uncertain (RCT, MA, GL)
**Secondary stroke prevention**	▲ In patients with prior ICH, BP control to ≤ 130/80 mmHg reduces the risk of subsequent stroke; polypill combination therapy is a proven strategy to achieve this goal (RCT) ▼ In patients with ESUS, DOACs are not superior to aspirin for secondary prevention and may increase bleeding; antiplatelet therapy remains first-line treatment (MA) ▲ In AF patients, early DOAC initiation within 4 days after acute ischaemic stroke should be considered (IPDMA). Adding antiplatelet therapy to anticoagulation is not supported in AF patients with concomitant atherosclerotic cardiovascular disease (RCT) ▲ Asundexian, an oral factor XIa inhibitor, represents a novel promising treatment strategy to reduce recurrent stroke without excess bleeding in patients with non-cardioembolic ischaemic stroke or high-risk TIA (RCT) • More data are needed for aetiology-specific treatments, including anti-inflammatory therapy (MA), and left atrial appendage occlusion (RCT)
**Stroke in the young and less common causes**	• Both traditional risk factors (obesity, hypertension, diabetes, low HDL cholesterol) and non-traditional risk factors (migraine with aura, psychological stress) have been associated with young-onset ischaemic stroke, supporting a broad risk-factor assessment in clinical practice (OBS) ▲ In CVT patients, DOACs represent a reasonable alternative to vitamin K antagonists (OBS) • In patients with CeAD, both anticoagulants and antiplatelets can be considered, with treatment tailored to individual patient profiles (OBS). Pregnancy is generally safe with postpartum vigilance (OBS)
**Outcomes and prognostication**	• Substantial residual stroke recurrence risk persists years after initial event, even with optimal secondary prevention (OBS) ▼ Routine prophylactic anti-seizure medication has not demonstrated benefit for seizure prevention (RCT) ▲ The Edinburgh Risk Score incorporating APOE genotyping predicts ICH, and carotid plaque imaging with high-resolution MRI (IMPROVE score) improves risk stratification for recurrent ischaemic stroke (OBS)
**Neurorehabilitation**	• Electromagnetic network targeting field therapy seems safe, and shows some promising trends in motor function recovery (RCT) ▲ ESO guidelines for post-stroke motor rehabilitation recommend ≥ 20 h of additional repetitive upper limb practice and high-intensity gait training to improve walking endurance (GL) ▲ Home-based, accessible rehabilitation programmes may improve self-efficacy and daily function (RCT)
**Post-stroke cognitive impairment and dementia**	• Metabolic syndrome is a key modifiable risk factor for late-onset post-stroke cognitive impairment, with diabetes and low HDL cholesterol showing the strongest associations (OBS). Newer agents such as GLP-1 receptor agonists warrant further investigation in RCTs ▲ Acute reperfusion therapies reduce post-stroke dementia risk by approximately two-thirds, underlining the importance of timely stroke treatment (OBS) • Emerging therapeutic approaches such as computerised cognitive training (MA), cardiorespiratory training (RCT) and brain stimulation (MA) appear promising and should be investigated in adequately powered RCTs
**Systems of care and implementation**	▲ MSUs improve functional outcome and are cost-effective in high-volume systems (RCT, OBS) ▲ Behaviour-change interventions can reduce door-to-puncture times and improve 90-day outcomes for EVT (RCT) • Personalised prehospital triage algorithms may optimise routing of LVO patients (OBS)
**Others**	▼ Recent randomised evidence does not support routine IVT for acute central retinal artery occlusion (RCT); enrolment of patients in future RCTs is encouraged ▼ Left atrial appendage occlusion appears not to benefit high-risk AF compared with medical therapy in primary stroke prevention (RCT) ▲ For asymptomatic high-grade carotid stenosis, adding stenting to intensive medical management may benefit selected patients over medical management alone (RCT)

Abbreviations: AF, atrial fibrillation; APOE, apolipoprotein E; BP, blood pressure; CeAD, cervical artery dissection; CVT, cerebral venous thrombosis; DOAC, direct oral anticoagulant; ESUS, embolic stroke of undetermined source; GLP-1, glucagon-like peptide-1; HDL, high-density lipoprotein (cholesterol); IVT, intravenous thrombolysis; MSU, mobile stroke unit; SBP, systolic blood pressure; Xa, factor Xa; MA meta-analysis; IPDMA, individual patient data meta-analysis; OBS, observational study; GL, guideline.

However, important questions remain. The role of IA thrombolytic therapy following successful EVT and surgical approaches for ICH require further investigation. We have yet to establish the optimal role of rhythm control strategies in secondary stroke prevention, and further research is needed to identify effective therapeutic approaches for AF patients who experience breakthrough stroke. Young stroke populations need better-defined antithrombotic strategies, including long-term, and effective interventions for PSCI are still lacking. Multiple ongoing RCTs are addressing these gaps and will inform future ESO guideline iterations.

Regular evidence synthesis has become essential as stroke medicine advances rapidly. This annual update format allows timely dissemination of practice-relevant findings while acknowledging current limitations and identifying research priorities. The ESO Guideline Board remains committed to this initiative as a means of supporting clinicians in delivering evidence-based stroke care and improving patient outcomes worldwide.
